# Pharmacodynamic monitoring by residual gene expression of the nuclear factor of activated T cell-regulated genes in lung transplant recipients and its correlation with tacrolimus blood levels

**DOI:** 10.3389/fimmu.2024.1382459

**Published:** 2024-05-10

**Authors:** Meritxell Boada-Pérez, Victoria Ruiz de Miguel, Marta Erro, Piedad Ussetti, Myriam Aguilar, Raquel Castejón, Silvia Rosado, Roser Escobar-Fornieles, Eva Revilla-López, Carlos Bravo, Berta Sáez-Giménez, Marta Zapata-Ortega, Yolanda Villena-Ortiz, Jaume Vima-Bofarull, Víctor Monforte, Susana Gómez-Ollés

**Affiliations:** ^1^ Department of Medicine, Universitat Autònoma de Barcelona, Barcelona, Spain; ^2^ Department of Pulmonology, Vall d’Hebron Institut de Recerca, Barcelona, Spain; ^3^ Department of Pulmonary Medicine, University Hospital Puerta de Hierro, Madrid, Spain; ^4^ Internal Medicine Laboratory, Instituto de Investigación Puerta de Hierro Segovia de Arana, Madrid, Spain; ^5^ Department of Pulmonology, Lung Transplant Program, Hospital Universitari Vall d’Hebron, Barcelona, Spain; ^6^ Centro de Investigación Biomédica en Red de Enfermedades Respiratorias (CIBERES), Instituto de Salud Carlos III, Madrid, Spain; ^7^ Department of Cellular Biology, Physiology and Immunology, Universitat Autònoma de Barcelona, Barcelona, Spain; ^8^ Central Laboratory Services, Pharmacology Section, Hospital Universitari Vall d’Hebron, Barcelona, Spain

**Keywords:** pharmacodynamics, pharmacokinetics, tacrolimus, rejection, infection, therapeutic drug monitoring, biomarker

## Abstract

**Introduction:**

Trough blood levels (C_0_) of tacrolimus are used to adjust drug dosage, but they do not consistently correlate with clinical outcomes. Measurement of residual gene expression of nuclear factor of activated T cell (NFAT)-regulated genes (NFAT-RGE) has been proposed as a pharmacodynamic biomarker to assess the degree of immunosuppression in certain solid organ transplantations, but little is known regarding lung transplant recipients (LTR). Our primary objective is to correlate tacrolimus blood levels with NFAT-RGE.

**Methods:**

NFAT-RGE and tacrolimus C_0_ and peak (C_1.5_) levels were determined in 42 patients at three, six and 12 months post-transplantation.

**Results:**

Tacrolimus C_0_ did not exhibit a correlation with NFAT-RGE, whereas C_1.5_ did. Besides, over 20% of measurements indicated high levels of immunosuppression based on the below 30% NFAT-RGE threshold observed in many studies. Among those measurements within the therapeutic range, 19% had an NFAT-RGE<30%.

**Conclusion:**

Consequently, a subset of patients within the tacrolimus therapeutic range may be more susceptible to infection or cancer, potentially benefiting from NFAT-RGE and tacrolimus peak level monitoring to tailor their dosage. Further quantitative risk assessment studies are needed to elucidate the relationship between NFAT-RGE and the risk of infection, cancer, or rejection.

## 1 Introduction

After lung transplantation (LT), immunosuppressive regimens are used to reduce the rate of acute and chronic rejection, which continue to represent significant sources of morbidity and mortality in this population (1). While immunosuppressive therapy protocols vary from center to center, conventional maintenance therapy consists of a triple drug combination with a calcineurin inhibitor (cyclosporine or tacrolimus, CNI), an antiproliferative agent, and corticosteroids (2). CNIs remain the cornerstone of immunosuppression following LT, and tacrolimus is currently the drug of choice. However, tacrolimus is characterized by a wide intra- and inter-individual pharmacokinetic variability and a narrow therapeutic index. Thus, therapeutic drug monitoring (TDM) is mandatory. In current clinical practice, tacrolimus dose adjustment is performed solely through a pharmacokinetic measurement of its trough levels, and in particular cases by performing an abbreviated area under the curve assessment. Nevertheless, pharmacokinetic monitoring does not have an optimal correlation with clinical outcomes (3), making it challenging to determine an individual patient’s risk for infection or rejection. Therefore, efforts are being made to find non-invasive biomarkers and pharmacodynamic monitoring strategies capable of measuring an individual patient’s state of immunosuppression.

One of the most promising immune function assays is the measurement of residual expression of nuclear factor of activated T-cells (NFAT)-regulated genes (NFAT-RGE) in the peripheral blood by real-time quantitative polymerase chain reaction (RT-qPCR) (3–12). The immunosuppressive effect of tacrolimus is produced by inhibition of the calcineurin-NFAT signaling pathway in T-helper cells. In broad terms, tacrolimus binds to an immunophilin causing a non-competitive inhibition of calcineurin-phosphatase activity which is necessary for activation of cytoplasmic nuclear factors such as NFAT, which then translocate to the nucleus and activate cytokine transcription, a critical step in T-cell activation (13). Among other actions, NFAT facilitates transcription of interleukin-2 (IL-2), interferon gamma (IFN-γ), or granulocyte and macrophage colony-stimulating factor (GM-CSF) (14, 15).

Most of the studies published so far on the use of NFAT-RGE refer to liver and kidney transplantation (3, 4, 8, 9, 12), and some of them have been performed in patients receiving cyclosporine A-based immunosuppression (6, 7, 9, 11, 16). Those studies have shown a lower residual expression among patients with recurrent infections or viremia and an increased risk of malignancies. In contrast, patients with a high residual cytokine expression have been associated with an increased risk of rejection (17). Several clinical trials have also demonstrated the feasibility of adjusting tacrolimus dosing according to NFAT-RGE in kidney and liver transplantation (8, 18). However, there is still little information on the use of this promising pharmacodynamic tool in the field of lung transplantation. Though information on NFAT-RGE assessment in LT recipients (LTR) is scarce, it has been tentatively suggested that the biomarker may be useful in identifying recipients at an increased risk of infection (19).

The aim of this study is to assess NFAT-RGE in relation to trough and peak blood levels of tacrolimus in LTR. We also aim to stratify patients by their grade of immunosuppression based on the below 30% NFAT-RGE threshold observed in many studies.

## 2 Materials and methods

### 2.1 Study design and population

This prospective, observational study included 42 LTR transplanted between February 2020 and September 2021. Patients were over 18 years old and were willing to participate in the study during the first 12 months post-transplant, all providing written informed consent. All procedures were conducted in accordance with the ethical standards of Vall d’Hebron University Hospital’s ethics committee (PR(AG)101/2019), and with the Declaration of Helsinki (2013).

All patients were enrolled before transplantation, and blood samples were collected at three (33 samples), six (33 samples) and 12 (21 samples) months after transplantation concurrently with routine blood tests. Ten patients contributed only one sample, 19 provided two samples and 13 patients had samples collected at all three time points.

Patients received immunosuppressive therapy according to institutional protocols, which included a triple-drug regimen consisting of tacrolimus, mycophenolic acid, and corticosteroids. In some cases, mycophenolic acid had to be temporarily discontinued due to leukopenia. The target trough levels for tacrolimus were set between 10-15 ng/mL. All patients initially received immediate-release tacrolimus, and five required a switch to extended-release tacrolimus. Thus, a total of 82 samples were collected with immediate-release tacrolimus, and five samples with extended-release tacrolimus.

### 2.2 Tacrolimus pharmacokinetic monitoring

Tacrolimus trough (C_0_) and peak (C_1.5_) levels were analyzed using high-performance liquid chromatography-mass spectrometry (HPLC-MS) at the clinical laboratories of Vall d’Hebron University Hospital.

### 2.3 Tacrolimus pharmacodynamic monitoring

The blood samples used for pharmacodynamic monitoring were obtained in parallel with the blood samples for pharmacokinetic monitoring and were analyzed at Vall d’Hebron Research Institute.

To do so, blood samples were collected in BD Vacutainer^®^ lithium heparin tubes. One milliliter of heparinized blood was stimulated with 1 mL of complete RPMI 1640 (Gibco) enriched with 100 ng/mL of phorbol 12-myristate 13-acetate (PMA) and 5 µg/mL of ionomycin (Sigma Aldrich, Saint Louis, MO) for three hours at 37°C and 5% CO_2_. Then, 10 mL of RNA/DNA Stabilization Reagent for Blood/Bone Marrow (Roche, Mannheim, Germany) were added to lyse cells and stabilize nucleic acids, according to the manufacturer’s instructions. Samples were stored at -20°C for a maximum of a year.

mRNA was isolated using an mRNA Isolation Kit for Blood/Bone Marrow (Roche, Mannheim, Germany) following the manufacturer’s instructions. It was then stored at -20°C for a week or less, or at -80°C indefinitely.

Reverse transcription was performed with 8.2 µL of mRNA in a thermocycler using avian myeloblastosis virus reverse transcriptase and oligo (dT) as a primer (First Strand cDNA synthesis kit Roche, Mannheim, Germany) following the manufacturer’s instructions. The resulting cDNA was stored concentrated at -20°C until further use.

### 2.4 Gene expression analysis

First, cDNA was diluted to a final volume of 200 µL by adding 180 µL of water. Gene expression of three genes regulated by NFAT (IL-2, IFN-γ and GM-CSF) was quantified by performing real-time quantitative PCR in a LightCycler^®^ 480 thermocycler (Roche Diagnostics, Basel, Switzerland). Target sequences were amplified using commercially available LightCycler Primer Sets (Search-LC, Heidelberg, Germany) with the LightCycler FastStart DNA Sybr Green I Kit (Roche Diagnostics, Basel, Switzerland), following the manufacturer’s instructions. Transcript concentrations for each gene were calculated from a virtual standard curve. The mRNA input was normalized by the constant expression of two reference genes (β-actin and cyclophilin B). Quantification cycle values were determined using the fit point method.

The percentage of NFAT-RGE was calculated as: [(IL-2 adjusted transcripts C_1.5_/C_0_ X 100) + (IFN-γ adjusted transcripts C_1.5_/C_0_ X 100) + (GM-CSF adjusted transcripts C_1.5_/C_0_ X 100)]/3. For further details, see previous reports (4, 6, 9, 19).

Previous studies, mostly conducted in kidney transplantation, demonstrated that patients with an NFAT-RGE lower than 30% were at high risk of infection and malignancies (5). Therefore, this study used a cut-off of NFAT-RGE <30% to stratify patients as having a high degree of immunosuppression and an increased risk of infection.

### 2.5 Statistical analysis

Continuous variables were presented as mean ± standard deviation (SD) or median ± interquartile range (IQR). Categorical variables were described as frequencies and percentages. Normality of distributions was evaluated before performing statistical analyses to determine the most appropriate test for each case. Chi-Square or Fisher’s exact test was used for comparisons between qualitative variables, while t-tests for two independent variables/ANOVA test (>2 categories) or Mann Whitney/Kruskal Wallis U tests were employed for continuous variables. Pearson’s test was used to examine linear correlations in Gaussian variables, and Spearman’s was used for non-Gaussian variables. Statistical significance was set at p ≤ 0.05. All analyses were performed using Graphpad Prism 9.0 statistical software (GraphPad Software, LLC, San Diego, CA).

## 3 Results

### 3.1 Inhibition of NFAT-regulated genes over time after LT

**Figure 1:**
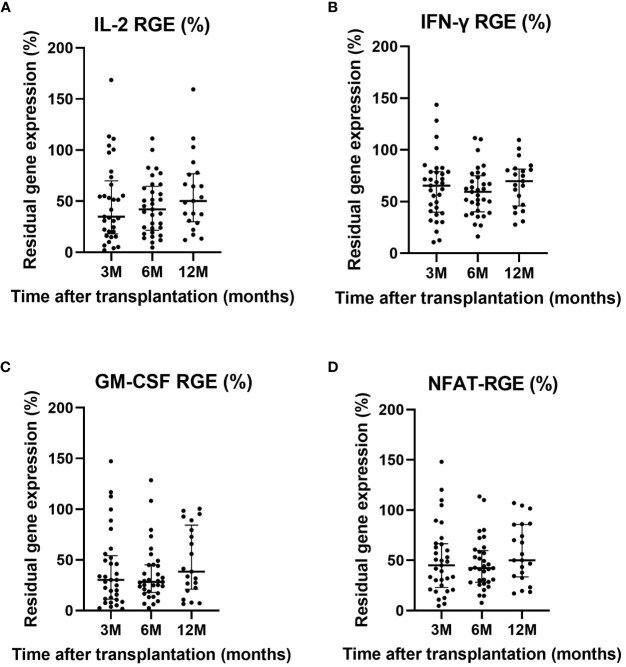
Residual gene expression of IL-2 **(A)**, IFN-γ **(B)**, GM-CSF **(C)** and NFAT-RGE **(D)** at three, six and 12 months after LT. Only significant p-values are shown (p<0.05).

Residual expression of the three NFAT-regulated genes analyzed in this project remained stable over time after LT. The median IL-2 RGE was 35% (IQR=18%-70%) at three months, 42% (IQR=22%-65%) at six months, and 50% (IQR=30%-77%) after one year (**Figure 1A**). The median IFN-γ RGE was 65% (IQR=40%-79%) at three months, 59% (IQR=40%-75%) at six months, and 70% (IQR=46%-81%) after one year (**Figure 1B**). The median GM-CSF RGE was 30% (IQR=11%-54%) at three months, 28% (IQR=18%-45%) at six months, and 38% (IQR=21%-84%) after one year (**Figure 1C**). Overall, the median NFAT-RGE was 45% (IQR=23%-66%) at three months, 42% (IQR=28%-60%) at six months, and 50% (IQR=33%-86%) after one year (**Figure 1D**).

Approximately 30% (n=9) of patients at three months, 30% (n=9) at six months, and 19% (n=9) at 12 months were considered to have high-grade immunosuppression based on NFAT-RGE analysis.

Comparison of clinical, demographic, and therapeutic characteristics between patients with overall high-grade immunosuppression (NFAT-RGE <30%), calculated as the median value of all time-point determinations, and overall low-grade immunosuppression (NFAT-RGE ≥30%), are shown in **Table 1**. Strikingly, no differences were observed in C_0_ tacrolimus levels (9.3 vs, 10.3, p = 0.265), but doses of tacrolimus (8.5 vs. 6, p<0.001) and C_1.5_ levels (31.2 vs. 20.7, p<0.001) differed, being higher in patients with an NFAT-RGE <30%. As expected, residual gene expression of each of the three different cytokines regulated by NFAT was significantly lower when NFAT-RGE was below 30%. GM-CSF residual gene expression was always the lowest, followed by IL-2 and IFN-ɣ (**Table 1**).

**Table 1:** Clinical and demographic characteristics of the population, stratifying patients into high or low-grade immunosuppression according to their overall NFAT-RGE.

Characteristic	All patients	Overall high-grade immunosuppression <30% NFAT-RGE N=10	Overall low-grade immunosuppression ≥30% NFAT-RGE N=32	P value
**Male gender n (%)**	27 (64)	2 (20)	25 (78)	**0.002**
**Age (years) median (IQR)**	59 (31-68)	61 (58-65)	55 (47-61)	0.090
**BMI (kg/m^2^) median (IQR)**	23 (21-25)	22 (20-27)	23 (21-25)	0.787
**Double-LT n (%)**	39 (93)	8 (80)	31 (97)	0.136
**Underlying lung disease** n **(%)**				
** Interstitial lung disease**	22 (52)	4 (40)	18 (56)	0.716
** COPD**	14 (33)	5 (50)	9 (28)	
** Cystic fibrosis**	4 (9)	1 (10)	3 (9)	
** Bronchiectasis**	1 (2)	0 (0)	1 (3)	
** Pulmonary hypertension**	1 (2)	0 (0)	1 (3)	
**Corticosteroid doses (mg/day)** **median (IQR)**	16 (15-24)	20 (16-21)	16 (12-24)	0.213
**Mycophenolic acid doses (mg/day) median (IQR)**	720 (360-1000)	1000 (360-1000)	720 (360-1000)	0.275
**Tac doses (mg/day) median (IQR)**	6.5 (5-8)	8.5 (6-10)	6 (4-8)	**<0.001**
**Tac C_0_ (ng/mL) median (IQR)**	10.3 (8-12)	9.3 (8-11)	10.3 (8-12)	0.264
**Tac C_1.5_ (ng/mL) median (IQR)**	23.2 (15-31)	31.2 (24-35)	20.7 (14-26)	**<0.001**
**IL-2-RGE (%) median (IQR)**	42 (22-67)	16 (9-20)	54 (36-78)	**<0.001**
**IFN-γ-RGE (%) median (IQR)**	62 (41-79)	34 (28-39)	71 (60-82)	**<0.001**
**GM-CSF-RGE (%) median (IQR)**	30 (17-56)	8 (6-13)	38 (28-73)	**<0.001**

IQR, Interquartile range; BMI, body mass index; LT, lung transplantation; COPD, Chronic obstructive pulmonary disease; NFAT-RGE, nuclear factor of activated T cells residual gene expression; Tac, tacrolimus; Comparisons between groups, Mann–Whitney U-test for continuous variables; Chi-squared test for categorical variables. Bold value: statistically significant result (p<0.05).

### 3.2 Tacrolimus pharmacokinetics

**Figure 2:**
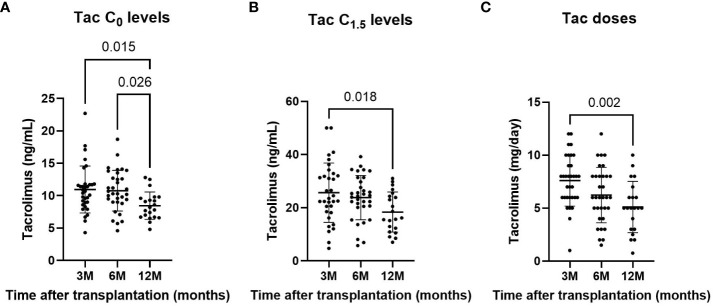
Tacrolimus C_0_ **(A)** and C_1.5_ **(B)** concentrations, and tacrolimus doses **(C)** at three, six and 12 months after LT. Only significant p-values are shown. significant result (p<0.05)

The overall mean tacrolimus C_0_ levels were 10.3 ng/mL ± 3.3 and decreased over time. At three months after LT, mean tacrolimus C_0_ levels were 10.9 ng/mL ± 3.6, 10.8 ng/mL ± 3.1 at six months, and 8.5 ng/mL ± 2.11 at 12 months (**Table 2**). Tacrolimus C_0_ levels significantly decreased at 12 months compared to three months (p=0.015) and six months (p=0.026) (**Figure 2A**).

**Table 2:** Tacrolimus mean concentrations (C_0_ and C_1.5_) and doses at 3, 6, 12 months after transplantation and all time points.

	All time points N = 87 determinations	3 months N = 33 determinations	6 months N = 33 determinations	12 months N = 21 determinations
**Tac C_0_** **(ng/mL) mean ( ± SD)**	10.3 ± 3.3	10.9 ± 3.6	10.8 ± 3.1	8.5 ± 2.1
**Tac C_1.5_** **(ng/mL) mean ( ± SD)**	23.2 ± 9.7	25.7 ± 11.2	23.8 ± 8.3	18.4 ± 7.5
**Tac doses** **(mg/day) mean ( ± SD)**	6.6 ± 2.7	7.6 ± 2.4	6.2 ± 2.6	5.1 ± 2.4

Tac, tacrolimus; SD, Standard deviation; C_0_, Tacrolimus trough or predose concentration; C_1.5_, Tacrolimus concentration 1.5 h postdose.

The overall mean tacrolimus C_1.5_ level was 23.2 ng/mL ± 9.7 and tended to decrease over time. At three months after LT, mean C_1.5_ levels were 25.7 ng/mL ± 11.2, 23.8 ng/mL ± 8.3 at six months, and 18.4 ng/mL ± 7.5 at 12 months. Tacrolimus C_1.5_ levels were significantly lower at 12 months than at three months (p=0.018) (**Figure 2B**).

The overall mean tacrolimus dose was 6.5 mg/day ± 2.7 and also decreased over time. At three months after LT, mean doses were 7.6 mg/day ± 2.4, 6.2 mg/day ± 2.6 at six months, and 5.1 mg/day ± 2.4 at 12 months. Tacrolimus doses were significantly lower at 12 months than at three months (p=0.002) (**Figure 2C**).

Almost 60% (n=51) of the measurements were outside the tacrolimus therapeutic range (10-15 ng/mL); of those, 12% (n=6) were above the range. Approximately 20% of the measurements that would today be considered within the therapeutic range actually represented a high degree of immunosuppression according to NGAT-RGE. Surprisingly, NFAT-RGE <30% was found in only one out of six measurements above the therapeutic range (**Table 3**).

**Table 3:** Classification of determinations based on their NFAT-RGE and tacrolimus C_0_ concentrations.

	All determinations	NFAT-RGE<30% (n=22)	NFAT-RGE≥30% (n=65)
**Tac C_0_<10 ng/mL n (%)** **Under-immunosuppression**	45 (52)	14 (31)	31 (69)
**Tac C_0_ 10-15 ng/mL n (%)** **Therapeutic range**	36 (41)	7 (19)	29 (81)
**Tac C_0_>15 ng/mL n (%)** **Over-immunosuppression**	6 (7)	1 (17)	5 (83)

Tac, tacrolimus; C_0_, tacrolimus trough or predose concentration; C_1.5_, Tacrolimus concentration 1.5 h postdose.

### 3.3 NFAT-RGE and tacrolimus pharmacokinetics

**Figure 3:**
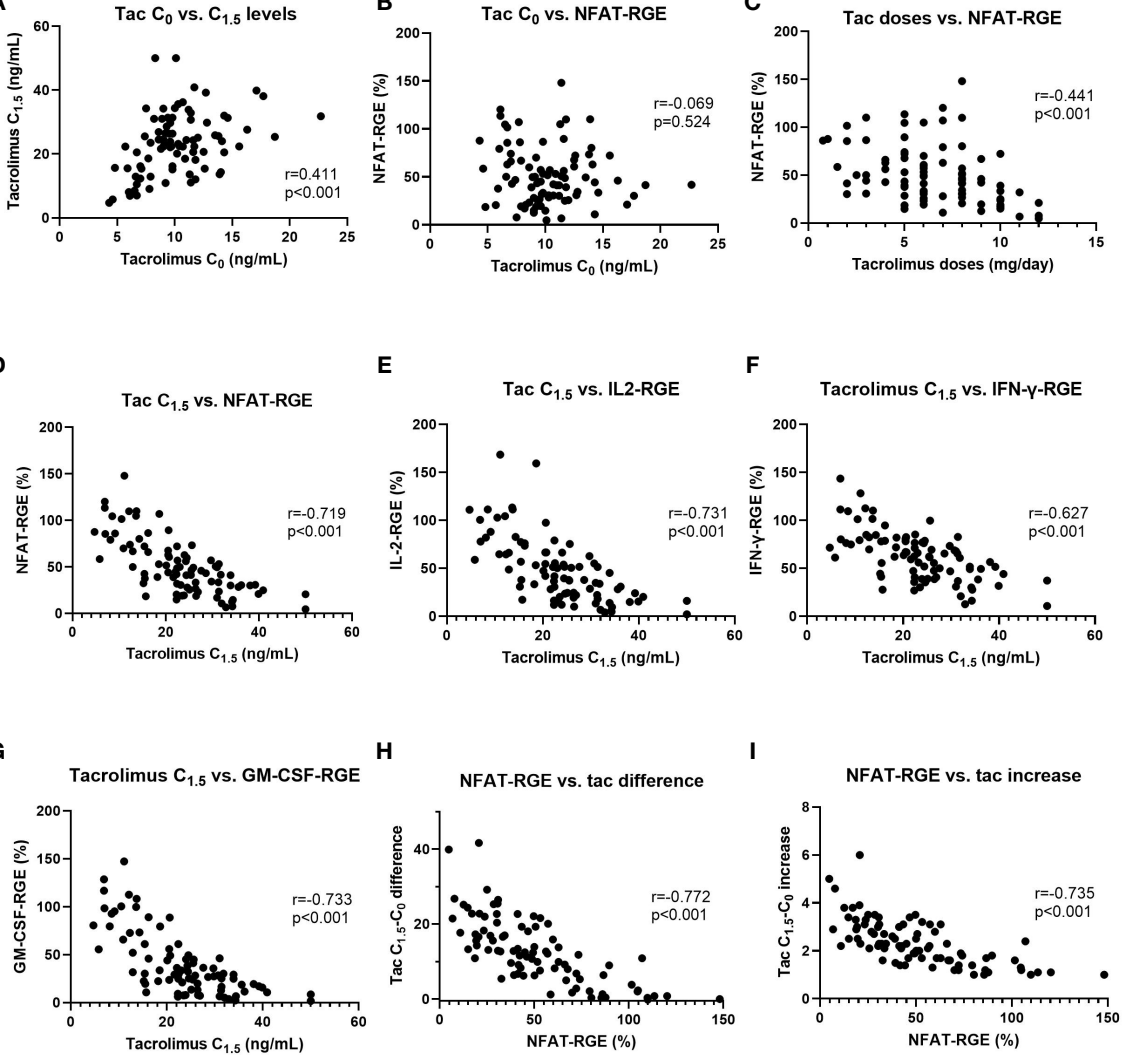
Linear correlations between tacrolimus C_0_ vs. C_1.5_ **(A)**, tacrolimus C_0_ vs. NFAT-RGE **(B)**, tacrolimus doses vs. NFAT-RGE **(C)**, tacrolimus C_1.5_ vs. NFAT-RGE **(D)**, tacrolimus C_1.5_ vs. IL2-RGE **(E)**, tacrolimus C_1.5_ vs. IFNγ-RGE **(F)**, tacrolimus C_1.5_ vs. GM-CSF-RGE **(G)**, NFAT-RGE vs. tacrolimus difference (C_1.5_-C_0_) **(H)**, and NFAT-RGE vs. tacrolimus increase (ratio calculated as C_1.5_/_C0_) **(I)**.

Tacrolimus C_0_ levels showed a moderate correlation with C_1.5_ levels (r=0.410, p<0.001) (**Figure 3A**). Tacrolimus C_0_ levels did not correlate with any of the three NFAT-regulated genes, analyzed either separately or overall (r=-0.069, p=0.524) (**Figure 3B**). Tacrolimus doses showed a moderate correlation with NFAT-RGE analyzed separately or overall (r=-0.441, p<0.001) (**Figure 3C**).

Nevertheless, NFAT-RGE showed a strong negative correlation with tacrolimus C_1.5_ levels both overall (r=-0.719, 95% CI -0.8095 to -0.5950 p<0.001) (**Figure 3D**) and separately: IL-2 (r=-0.731, 95% CI -0.818 to -0.611, p<0.001) (**Figure 3E**), IFN-γ (r=-0.627, 95% CI -0.743 to -0.475, p<0.001) (**Figure 3F**), and GM-CSF (r=-0.733, 95% CI -0.819 to -0.613, p<0.001) (**Figure 3G**).

Moreover, NFAT-RGE presented a negative correlation with the tacrolimus difference measured as C_1.5_-C_0_ (r=-0.772, 95% CI -0.847 to -0.666, p<0.001) (**Figure 3H**) or as an increase (ratio), C_1.5_/C_0_ (r=-0.735, 95% CI -0.821 to -0.616, p<0.001) (**Figure 3I**).

### 3.4 NFAT-RGE and its correlation with corticosteroids and mycophenolic acid

**Figure 4:**
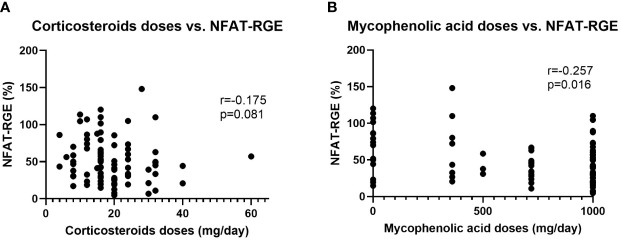
Linear correlations between corticosteroid doses vs. NFAT-RGE **(A)** and mycophenolic acid doses vs. NFAT-RGE **(B)**.

When classifying patients according to their NFAT-RGE, there were no statistically significant differences in corticosteroid or mycophenolic acid doses (**Table 1**). Nor was there a correlation between corticosteroid doses (r=-0.175, 95% CI -0.377 to 0.043, p=0.081) (**Figure 4A**) or mycophenolic acid doses (r=-0.257, 95% CI -0.4491 to -0.043, p=0.016) (**Figure 4B**) and NFAT-RGE.

However, when we split the determinations between those taken under methylprednisolone (n=11) and prednisone (n=76), we observed a very strong correlation between prednisone doses and NFAT-RGE (r=-0.84; p=0.002), but the correlation is non-existent when relating methylprednisolone and NFAT-RGE (r=-0.08; p=0.502).

## 4 Discussion

Despite tacrolimus being a cornerstone of immunosuppressive treatment in LTR, optimizing doses remains challenging due to its high inter- and intra-individual variability. Tacrolimus monitoring aims to balance efficacy, attempting to prevent chronic lung allograft dysfunction without increasing the risk of infections and malignancies. Currently, monitoring is based solely on tacrolimus trough blood levels, yet this pharmacokinetic measurement appears insufficient as it does not reflect the patient’s actual immunosuppressive state. Previous studies have demonstrated the usefulness of measuring NFAT-RGE in clinical trials monitoring kidney, liver, and heart transplant recipients (9, 11, 12). Moreover, this assay has proven highly reproducible and feasible in routine molecular biological laboratories (3, 9–11, 20), suggesting its potential integration into current clinical practice. However, the value of this pharmacodynamic tool in LTR remains unclear (19).

In our study, we observed that tacrolimus drug monitoring based on C_0_ blood levels does not correlate with the level of immunosuppression measured by NFAT-RGE; nearly 24% of patients had a median NFAT-RGE <30%. Consistent with previous studies, we also found that NFAT-RGE correlated better with tacrolimus peak concentrations than with trough concentrations (5, 19, 21).

NFAT-RGE levels below 30% generally indicate a high degree of immunosuppression, as an increased probability of infection and neoplasia has been observed (5, 8, 16, 22, 23). For instance, Sommerer et al. (5) demonstrated that patients with minimal or NFAT-RGE<30% had significantly more infections and malignancies, whereas patients with insufficient inhibition suffered more acute rejections. Similarly, in an LTR cohort, Greenland et al. (19) observed that pulmonary infections detected by bronchoscopy were more common with NFAT-RGE<40%.

Regarding graft rejection, most studies have focused on acute rather than chronic rejection episodes in other solid organ transplants. Sommerer et al. (5) showed that only 1.3% of patients with an NFAT-RGE <30% developed biopsy-proven acute rejection compared to 25.2% of those with NFAT-RGE >30%. Similarly, Millán et al. (24) demonstrated higher NFAT-RGE levels in patients with T cell-mediated acute rejection (75% [42-100%]) or subclinical cellular rejection (41% [18-78%]) compared with patients without these events (14% [2-23%]). Several other publications reported similar results with tacrolimus (3, 5) and or cyclosporine (8, 23). However, in the case of LTR, Greenland et al. (19) did not observe a correlation between higher NFAT-RGE levels and lung allograft acute rejection.

In our study, when classifying patients by their degree of immunosuppression according to NFAT-RGE results, corticoid or mycophenolic acid doses did not show significant differences. Moreover, there was no correlation between corticosteroid doses or mycophenolic acid doses and NFAT-RGE. According to Sommerer et al. (5), NFAT-RGE seems to be affected by tacrolimus but not by corticosteroids or mycophenolic acid. However, Greenland et al. (19) did observe a significant negative correlation between prednisone and NFAT-RGE. The differences observed between studies regarding the effect of corticosteroids on NFAT-RGE may be attributed to the specific type of corticosteroid administered to patients, as observed in our study when samples were categorized based on the type of corticosteroids used. In the study by Greenland et al. (19), patients were administered prednisone, whereas in our study, only 13% (n=11) of all determinations were analyzed under prednisone intake, with methylprednisolone being the most commonly used corticosteroid. Besides, in the study by Sommerer et al. (5), patients were administered methylprednisolone.

In accordance with the protocols, the doses of immunosuppressants are gradually reduced over time after transplantation. In our cohort, tacrolimus doses, as well as tacrolimus trough and peak blood levels, were significantly lower at 12 months compared to three months after LT. In the study by Sommerer et al. (3) tacrolimus doses in kidney transplant recipients increased from day 7 to month 2 and then stabilized. The difference could be explained by the lower tacrolimus trough levels required by kidney transplant recipients required compared to LTR.

In our study, the total residual expression of NFAT-RGE and of each gene individually increased over time, although not significantly, perhaps due to the changing sample size between 3-6 months and 12 months, or to the fact that the earliest time point tested was three months. In this context, Greenland et al. (19) observed in the early post-LT period that NFAT-RGE increased by 0.35% (95% CI 0.001-0.69%, p = 0.049) per week.

Regarding the therapeutic range of tacrolimus C_0_, almost 20% of the determinations in the therapeutic range in this study had an NFAT-RGE <30%, meaning that some patients with optimal trough tacrolimus concentrations were actually exposed to high-grade immunosuppression and might benefit from a dose reduction. Thus, adding the measurement of NFAT-RGE might improve drug monitoring.

The utility of NFAT-RGE as an add-on to pharmacokinetic drug monitoring with calcineurin inhibitor dose adjustment has already been explored in other solid organ transplants. Sommerer et al. (6) published a prospective, randomized clinical trial (1:1) in 54 stable patients after kidney transplant and observed that the group of patients whose cyclosporine dose was adjusted by NFAT-RGE monitoring had fewer infections, better renal function, and less arterial stiffness than those monitored in the conventional way (6). Similar studies in LT would be very useful. The main problem regarding their performance is the need to establish the optimal NFAT-RGE cut-off in this organ – that is to say, the grade of immunosuppression that presents an idea balance between the risk of infection and the risk of acute rejection or chronic graft dysfunction. This NFAT-RGE cut-off point will probably be different from that used in other types of transplants since the risk of infection in LT is significantly higher. In fact, Greenland et al. (19) found that the best cut-off in their LT cohort was 40% rather than the 30% used in all the other studies performed in kidney, liver and heart transplantation (9, 11, 12). More prospective studies with LTR stratifying the risk of infection and rejection according to NFAT-RGE determination seem necessary to confirm the optimal NFAT-RGE in LTR.

This study is part of a larger project assessing clinical outcomes 18 months after surgery. These data are no yet available; thus, one of the main limitations of this study is that at present we cannot link the NFAT-RGE results with the occurrence of infections in this population. Additionally, the sample size is small, and the data are derived from a single center.

Further clinical studies in larger LTR cohorts should assess whether this pharmacodynamic tool can predict patients at higher risk of developing infections, acute rejections, malignancies, and chronic lung allograft dysfunction, which is the main cause of graft loss within a year of lung transplantation and affects 41% of LTR patients within five years (25). Moreover, investigating whether NFAT-RGE determination could be useful in complex scenarios involving patients with recurrent infections taking apparently low immunosuppressant doses would be of interest.

## 5 Conclusions

Tacrolimus trough levels, currently used for therapeutic drug monitoring, did not correlate with the degree of immunosuppression measured by NFAT-RGE in LTR. However, peak levels showed a moderate correlation. A significant percentage of patients exhibited high levels of immunosuppression based on NFAT-RGE, despite being within the therapeutic range for tacrolimus. Incorporating NFAT-RGE measurement and tacrolimus peak levels into current pharmacokinetic monitoring may assist physicians in adjusting tacrolimus doses. Nevertheless, further studies are needed to establish optimal NFAT-RGE cut-offs in LTR to prevent infection and rejection events.

## Data availability statement

The raw data supporting the conclusions of this article will be made available by the authors, without undue reservation.

## Ethics statement

The studies involving humans were approved by Vall d’Hebron University Hospital’s ethics committee. The studies were conducted in accordance with the local legislation and institutional requirements. The participants provided their written informed consent to participate in this study.

## Author contributions

MB-P: Formal analysis, Investigation, Methodology, Writing – original draft, Writing – review & editing. VRM: Investigation, Writing – review & editing. ME: Investigation, Writing – review & editing. PU: Investigation, Writing – review & editing. MA: Investigation, Writing – review & editing. RC Investigation, Writing – review & editing. SR: Investigation, Writing – review & editing. RE-F: Investigation, Writing – review & editing. ER-L: Investigation, Writing – review & editing. CB: Investigation, Writing – review & editing. BS-G: Investigation, Writing – review & editing. MZ-O: Investigation, Writing – review & editing. YV-O: Investigation, Writing – review & editing. JV-B: Investigation, Writing – review & editing. VM: Conceptualization, Funding acquisition, Investigation, Methodology, Project administration, Supervision, Writing – original draft, Writing – review & editing. SG-O: Conceptualization, Formal analysis, Funding acquisition, Investigation, Methodology, Project administration, Supervision, Writing – original draft, Writing – review & editing.
